# Discrimination-based sample size calculations for multivariable prognostic models for time-to-event data

**DOI:** 10.1186/s12874-015-0078-y

**Published:** 2015-10-12

**Authors:** Rachel C. Jinks, Patrick Royston, Mahesh KB Parmar

**Affiliations:** MRC Clinical Trials Unit at UCL, Aviation House, 125 Kingsway, London, WC2B 6NH, UK

**Keywords:** Prognostic modelling, Sample size, Survival data, Multivariable models

## Abstract

**Background:**

Prognostic studies of time-to-event data, where researchers aim to develop or validate multivariable prognostic models in order to predict survival, are commonly seen in the medical literature; however, most are performed retrospectively and few consider sample size prior to analysis. Events per variable rules are sometimes cited, but these are based on bias and coverage of confidence intervals for model terms, which are not of primary interest when developing a model to predict outcome. In this paper we aim to develop sample size recommendations for multivariable models of time-to-event data, based on their prognostic ability.

**Methods:**

We derive formulae for determining the sample size required for multivariable prognostic models in time-to-event data, based on a measure of discrimination, *D*, developed by Royston and Sauerbrei. These formulae fall into two categories: either based on the significance of the value of *D* in a new study compared to a previous estimate, or based on the precision of the estimate of *D* in a new study in terms of confidence interval width. Using simulation we show that they give the desired power and type I error and are not affected by random censoring. Additionally, we conduct a literature review to collate published values of *D* in different disease areas.

**Results:**

We illustrate our methods using parameters from a published prognostic study in liver cancer. The resulting sample sizes can be large, and we suggest controlling study size by expressing the desired accuracy in the new study as a relative value as well as an absolute value. To improve usability we use the values of *D* obtained from the literature review to develop an equation to approximately convert the commonly reported Harrell’s *c*-index to *D*. A flow chart is provided to aid decision making when using these methods.

**Conclusion:**

We have developed a suite of sample size calculations based on the prognostic ability of a survival model, rather than the magnitude or significance of model coefficients. We have taken care to develop the practical utility of the calculations and give recommendations for their use in contemporary clinical research.

## Background

Prognosis is one of the central principles of medical practice. Understanding the likely course of a disease or condition is vital if clinicians are to treat patients with confidence or any degree of success. No two patients with the same diagnosis are exactly alike, and the differences between them – e.g. age, sex, disease stage, genetics – may have important effects on the course their disease will take. Such characteristics are called ‘prognostic factors’, and this phrase is usually taken to mean a factor which influences outcome independently of treatment.

For most applications, a single predictor is not sufficiently precise; rather a multivariable approach to prognosis is required. Multivariable prognostic research enables the development of tools which give predictions based on multiple important factors; these are variously called prognostic models, prediction models, prediction rules or risk scores [1]. Such research also means that potential new prognostic factors are investigated more thoroughly, as it allows the additional value of the factor, above and beyond that of existing variables, to be established [1].

The majority of prognostic research is done retrospectively, simply because results are obtained much more quickly and cheaply by using existing data. In their 2010 review, Mallett et al. [2] found that 68 % of the 47 prognostic studies using time-to-event data included were retrospective. Altman [3] conducted a review of publications which presented or validated prognostic models for patients with operable breast cancer, and found that of the 61 papers reviewed, 79 % were retrospective studies. Disadvantages to retrospective studies include missing data, a problem which in general cannot be mitigated by researchers. In addition, the assumption that data are missing at random may be implausible in such datasets, biasing results [4]. This is particularly true with stored samples, for example McGuire et al. [5] report that tumour banks usually contain a disproportionate number of samples from larger tumours, which may introduce bias. Existing datasets may also contain many more candidate variables than are really required to develop a good model, which can lead to multiple testing problems and a temptation to ‘dredge’ the data [6].

The best way to study prognosis is in a prospective study, which ‘enables optimal measurement of predictors and outcome’ [1]. However, a hurdle to designing good quality prognostic studies – whether prospective or retrospective – is ensuring that enough patients are included in order that the study has the required precision of results. In the second of a series of papers on prognosis research strategies, Riley et al. [7] stress that in particular, studies aiming to replicate or confirm prognostic factors should ‘incorporate a suitable sample size calculation to ensure adequate power to detect a prognostic effect, if it exists’. Sample size is always an important issue for clinical studies; however, little research has been performed which pertains specifically to the sample size requirements of multivariable prognostic studies. In his review of 61 publications concerning breast cancer models, Altman [3] found that none justified the sample size used; and for many it was impossible to discern the number of patients or events contributing to the final model. Mallett et al. [2] found that although 96 % of studies in their review of survival models reported the number of *patients* included in analyses, only 70 % reported the number of *events* – a key quantity for time-to-event data. In the same review, 77 % of the studies included did not give any justification for the sample size used. It is perhaps unsurprising that most papers reporting prognostic research do not justify the sample sizes chosen, as little guidance is available to researchers on how many patients should be included in prognostic studies.

Calculations based on the standard formula for the Cox proportional hazards (PH) model [8] are available for the situation where just one variable is of primary interest, but other correlated variables need to be taken into account in the analysis [9–11]. For the more common scenario where researchers wish to produce a multivariable prognostic model and all model variables are potentially equally important, basing sample size on the significance of numerous individual variables is likely to be an intractable problem. In this situation the most often cited sample size recommendation is the rule of ‘10 events per variable’ (EPV) which originated from two simulation studies [12, 13]. In these studies, exponential survival times were simulated for 673 patients from a real randomised trial with 252 deaths and 7 variables (36 EPV), and then the number of deaths were varied to reduce the EPV. The authors found that choosing a single minimum value for EPV was difficult but that results from studies having fewer than 10 EPV should be ‘cautiously interpreted’ in terms of power, confidence interval coverage and coefficient estimation for the Cox model. A later simulation study found that in ‘a range of circumstances’ having less than 10 EPV still provided acceptable confidence interval coverage and bias when using Cox regression, but did not directly consider the statistical power of analyses nor the variability of the estimates [14]. It is perhaps inevitable that these two papers are often cited to justify low sample sizes. Indeed, Mallett et al. [2] found in their review of papers reporting development of prognostic models in time-to-event data, that of the 28 papers reporting sufficient information to calculate EPV, 14 had fewer than 10 EPV.

In this paper, we take *multivariable prognostic model* to mean a model which is a linear combination of weighted prognostic factors. However when developing such a model, the individual covariate effects of the prognostic factors may not be of major interest. Instead the main aim is likely to be measuring the ability of the model to predict outcomes for future patients, or to discriminate between groups of patients. Copas [15] says that ‘ …a good predictor may include variables which are “not significant”, exclude others which are, and may involve coefficients which are systematically biased’. Thus basing sample size decisions on the significance of model coefficients alone may not result in the best prognostic model, as well as being complex when the model has multiple terms. Currently there seem to be very few sample size calculations or recommendations for developing or validating multivariable models which are based on the prognostic ability of a model, rather than the significance of its coefficients. During a literature search, few papers were retrieved which consider the issue from this angle. Smith, Harrell and Muhlbaier [16] used simulation to assess the error in survival predictions with increasing numbers of model covariates. Datasets of 250 and 750 subjects (64 and 185 events respectively) were drawn from an exponential distribution such that the average 5-year survival was 75 %. Cox models were fitted to the simulated data, with between 1 and 29 uniformly distributed covariates. The authors found that in both the 64 and 185 event datasets, 5-year survival predictions from the Cox models became increasingly biased upwards as the EPV decreased. In both datasets, the average error was below 10 % when EPV >10, and below 5 % when EPV >20. For ’sick’ subjects – those at high risk of death – higher EPVs were required: EPV >20 was required to reduce the expected error to 10 %. This work suggests that an EPV of 20 may be considered a minimum if accuracy of predictions are important, however as it is found within a National Institutes of Health report, it is not easily available and so seems to be seldom cited. Additionally, two papers considered the effect of sample size on Harrell’s *c* index. Ambler, Seaman and Omar [17] noted that the value of the *c* index increased with the number of events, however this issue was not the main focus of the publication and so investigation of this aspect was limited in scope. Vergouwe et al. [18] considered the number of events required for reliable estimation of the *c* index in logistic regression models and suggested that a minimum of 100 events and 100 non-events be used for external validation samples, which is likely to be higher than 10 EPV in many datasets. However being based on binary data, the results are not directly comparable to the sample size issue in prognostic models of time-to-event data.

In this paper we aim to develop calculations based on the prognostic ability of a model in time-to-event data, as quantified by Royston & Sauerbrei’s *D* measure of prognostic ability. We first describe the *D* statistic, and then present sample size calculations based on *D* for use in prognostic studies. Finally we give examples and describe suggested methods for increasing the practical usability of the calculations.

## Methods

### Royston & Sauerbrei *D* measure

There are various discrimination based measures of prognostic ability available for models of time-to-event data. The measure we have chosen to develop our calculations is Royston and Sauerbrei’s *D* measure [19], which has been shown to have many good properties which are described below [20]. The most commonly used measure of prognostic ability is probably Harrell’s *c* index [21], however this measure has some disadvantages: it is affected by censoring [22] and has a scale which can be difficult to interpret. Acknowledging the popularity and prevalence of the *c* index in the literature, we do consider the relationship between *c* and *D* to ensure our methods are more widely usable (see Section Appendix: simulation studies to test sample size calculations).

*D* measures prognostic ability by quantifying the separation in observed survival curves between subgroups of patients with differing predicted risks. *D* was developed in the Cox model framework and is based on risk ordering; thus *D* can be calculated whether the prognostic tool outputs a continuous prognostic index, prognostic groups, or is even a subjective rule. However, it is assumed that the prognostic index resulting from the model is Normally distributed (although this is an approximation in the case of a non-continuous prognostic index). The full derivation of *D* can be found in Royston and Sauerbrei’s original paper [19], but briefly:

$$D=\kappa \sigma^{\ast }, $$

where *σ*^∗^ is an estimate of the standard deviation of the prognostic index values (under the assumption of Normality) and $\kappa = \sqrt {8/\pi }\simeq 1.60$, a constant used to give a direct interpretation to *D*, as follows.

*D* has an intuitively appealing interpretation as the log hazard ratio between two equal-sized prognostic groups formed by dichotomising the prognostic index at its median. *D*’s interpretation as a log hazard ratio means that it can be translated to a hazard ratio between equally sized prognostic groups; so a *D* of 1 corresponds to a hazard ratio of *e*^1^=2.7 and *D*=2 to *e*^2^=7.4. This allows researchers familiar with hazard ratios of treatment effects (for example) to have some idea of the increase in risk across the prognostic index of the model for a particular value of *D*. As a log hazard ratio, *D* can theoretically take any value in the range (−*∞*,*∞*), but in real situations it is likely to be much closer to zero. A literature search for published values of *D* in a wide range of disease areas found that the highest value out of 101 reported was 3.44; the second highest was 2.8 [23]. *D*=0 implies that the selected model is useless for prediction, and *D*<0 may arise when a model fitted to one dataset is validated on another, indicating that the original model was flawed in some way. Additionally, *D* has a functional relationship with a measure of explained variation ${R_{D}^{2}}$ [19]. This relationship is important as most researchers will be more familiar with the 0–100 % range of *R*^2^ in linear regression.

As well as its interpretability and applicability to many types of prognostic model, *D* has many other properties which make it suitable for practical use. These include robustness to outliers, sensitivity to risk ordering, independence from censoring (provided the prognostic model has been correctly specified and the PI is approximately normally distributed), and an easily calculated standard error [19]. Also, since it takes into account the fit of the model to the outcome data, it can be used in a model validation context; a vital part of a good prognostic study. Working with ${R_{D}^{2}}$, Choodari-Oskooei et al. [20] found that it was sensitive to marked non-normality of the prognostic index, but despite this concluded that overall it was one of two best explained variation measures for quantifying predictive ability (along with Kent and O’Quigley’s $R_{\textit {PM}}^{2}$ [24]). *D* and ${R_{D}^{2}}$ can be calculated in Stata using the user-written str2d command [25].

### Sample size calculations

#### Introduction

To develop the required calculations we start from the results in Armitage and Berry’s book [26] (p186) for comparison of the means of two independent groups, with equal within-group variance. In this normal errors case, we consider two means $\overline {x}_{1}$ and $\overline {x}_{2}$ measured in populations of size *n*_1_ and *n*_2_ respectively, where *s*^2^ is the within-group variance of the response variable in both populations. The standard error of the difference in $\overline {x}_{1}$ and $\overline {x}_{2}$ is given by

$$SE(\overline{x}_{1}-\overline{x}_{2})=\sqrt{s^{2}\left(\frac{1}{n_{1}}+\frac{1}{n_{2}}\right) }. $$

From this, various sample sizes can be calculated. If *n*_1_, $\overline {x}_{1}$ and *s*^2^ are known, and it is desired that a difference of $\overline {x}_{1}-\overline {x}_{2}=\delta $ will be just significant at the required two-sided *α* level with power 1−*β*, then the sample size required in the second population is

(1) $$\begin{array}{*{20}l} n_{2}=s^{2}\left[\left(\frac{\delta}{z_{1-\alpha/2 }+z_{1-\beta}}\right)^{2} -\frac{s^{2}}{n_{1}}\right]^{-1},  \end{array} $$

where *z*_*x*_ is the *x*-quantile of the standard normal distribution.

We can also calculate sample size in a different way, basing it instead on the confidence interval of the estimated quantity *δ*. In order that the new estimate of $\overline {x}_{2}$ will have a 100(1−*α*) *%* confidence interval of half width *w*, the sample size required is

(2) $$\begin{array}{*{20}l} n_{2}=s^{2}\left[\left(\frac{w}{z_{1-\alpha }}\right)^{2} -\frac{s^{2}}{n_{1}}\right]^{-1}.  \end{array} $$

We can work from the same ideas to develop sample size calculations based on *D*, as this quantity is also normally distributed [23]. Consider the scenario where estimates of *D* and *SE*(*D*) are available from a previous study using the same model, and researchers wish to validate the estimate of *D* for the model in a new study. Let *D*_1_ be the value of *D* in the first study, ${\sigma _{1}^{2}}$ the variance of *D*_1_, and *e*_1_ the number of events in the first study. Let *D*_2_ be the *D* value in the (proposed) second study with *e*_2_ events, and ${\sigma _{2}^{2}}=var(D_{2})$. The standard error of *D*_1_−*D*_2_ is thus $\sqrt {{\sigma _{1}^{2}}+{\sigma _{2}^{2}}}$. As this does not explicitly include *e*_1_ and *e*_2_ we must make an assumption about the relationship between the variance of *D* and the number of events in the study in order to obtain sample size calculations.

#### The quantity *λ*

To develop the calculations required, we make a proportionality assumption. This is that for a given model with a certain ‘true’ value of *D*, the ratio of the variances ${\sigma _{1}^{2}}$, ${\sigma _{2}^{2}}$ of *D* in two datasets with differing numbers *e*_1_, *e*_2_ of events (but sampled from the same distribution of covariates) equals the reciprocal of the ratio of the corresponding numbers of events:

$$\frac{{\sigma_{1}^{2}}}{{\sigma_{2}^{2}}}=\frac{e_{2}}{e_{1}^{{}}}. $$

This is reasonable, since the variance of a statistic is inversely related to the information in the data, which in a censored time-to-event sample is plausibly represented by the number of events [27]. We have shown through simulation and resampling that this assumption does hold reasonably well; and the larger the dataset, the better it holds (see [23], Tables 4.1 – 4.2).

Under the proportionality assumption we can write $e_{1}{\sigma _{1}^{2}}=e_{2}{\sigma _{2}^{2}}=\lambda $, where *λ* is a model- and disease-specific structural constant which is incorporated in our calculations. We can either estimate *λ* by its value in a previous study (termed *λ*_*s*_), or use an approximation incorporating a value of *D* and the proportion of censoring (cens) in the dataset:

(3) $$ \lambda_{m}=c_{0}+c_{1}D^{1.9}+c_{2}(D\cdot\text{cens})^{1.3},   $$

where *c*_0_=2.66, *c*_1_=1.26, and *c*_2_=−1.65. This model was developed from simulated data and found to be reasonably accurate (see [23], Section 4.7.5).

Although our findings regarding *λ* are approximations, this seems a reasonable price to pay when first constructing a new method of planning prognostic studies. Prospective sample size calculations are by definition based on ‘guesstimated’ parameters, and these are not always checked post hoc, so in this respect we feel that the approximations made above are not inappropriate.

#### A note on the standard error of *D*

We have found that the default estimate of the standard error of *D* output by the str2d Stata command tends to underestimate the true value (see [23], Section 3.3 for full details). The negative bias increases the higher *D* is; for example, when *D*=0.8 simulation studies using different combinations of dataset size and proportion of censoring showed that the relative bias varied between 0 and –8 %, whereas when *D*=3.2, it varied from –17 % to –24 %. As an estimate of the standard error of *D* is required to obtain *λ*_*s*_, a downward bias in this quantity could reduce the required sample size and lead to underpowering.

We have found that using bootstrapping with 500 replications to obtain the standard error reduces the bias greatly; we observed a relative bias of –2 % (on average) with the bootstrap estimator when *D*=3.2 compared to -20 % using the default method [23]. The str2d command has a bootstrap standard error option and we recommend researchers use this method instead of the default estimate when calculating *λ*_*s*_, particularly when *D*≥2.

#### Obtaining the sample size calculations

By applying this proportionality assumption, we can now write the standard error of *D*_1_−*D*_2_ as $\sqrt {{\sigma _{1}^{2}}+\lambda /e_{2}}$ which, using the same rearrangement as above, leads us to the following two calculations. Firstly, to detect a difference in *D* between the first and second studies of *δ* with significance level *α* and power 1−*β*:

(A) $$\begin{array}{*{20}l} e_{2}& =\lambda\left[ \left(\frac{\delta }{zz}\right)^{2}-{\sigma_{1}^{2}}\right]^{-1},  \end{array} $$

where *zz*=*z*_1−*α*/2_+*z*_1−*β*_ for a two-sided (superiority) *α* and *zz*=*z*_1−*α*_+*z*_1−*β*_ for a one-sided (non-inferiority) test. Secondly, in order that the estimate of *D*_1_−*D*_2_ has a 100(1−*α*) *%* confidence interval of half width *w*

(C) $$\begin{array}{*{20}l} e_{2}& =\lambda\left[ \left(\frac{w }{z_{1-\alpha }}\right)^{2}-{\sigma_{1}^{2}}\right]^{-1}.  \end{array} $$

By comparing (A) and (C) with (1) and (2) we can see there is an analogy between the common within-sample variance *s*^2^ and the quantity *λ*.

Note that unlike in typical sample size calculations, here the value of ${\sigma _{1}^{2}}$ is available from the first study. Since *e*_2_ must be positive, this places a lower limit on *δ* and *w* for these calculations: *δ*>*σ*_1_*zz*, and *w*>*σ*_1_*z*_1−*α*_. Having calculated minimum *δ* for various datasets, we feel that in general (A) and (C) are not very useful in practice and so do not consider them further. Instead we develop slightly different calculations which are described below.

### Significance based calculations

Instead of estimating a value of *D*_1_ and its standard error from a previous study, we pick a fixed target value of *D* that we call *D*^∗^ and assume this has zero uncertainty; so ${\sigma _{1}^{2}}=0$. Thus (A) becomes

(B) $$ e_{2}=\lambda \left(\frac{\delta }{zz}\right)^{-2}   $$

We further obtain two calculations from (B) which are defined by how *λ* is estimated. Substituting *λ*_*s*_ into (B) gives us (B1), while substituting *λ*_*m*_ gives us (B2):

(B1) $$ e_{2}=\lambda_{s}\left(\frac{\delta }{zz}\right)^{-2}   $$

(B2) $$ e_{2}=\lambda_{m}\left(\frac{\delta }{zz}\right)^{-2}.   $$

For a one-sided test *H*_0_:*D*^∗^−*D*_2_≥*δ* and *H*_*A*_:*D*^∗^−*D*_2_≤*δ*. For a two-sided test *H*_0_:*D*^∗^−*D*_2_=*δ* and *H*_*A*_:*D*^∗^−*D*_2_≠*δ*. If a previous study does exist, then either (B1) or (B2) can be used. If no previous study exists, then (B1) cannot be used as *λ*_*s*_ cannot be calculated. When using (B1) and (B2) *δ* has a lower bound of zero.

One major benefit of using *λ*_*m*_ is that using this approximation, different values of *D* and *cens* can be input which enables calculation of a range of sample sizes. This may be helpful in study planning where the value of *D* and likely censoring proportion in the new study is uncertain.

### Confidence interval based calculations

We can alter calculation (C) under the same assumption of a fixed target *D* which we call *D*^∗^, as for (B1) and (B2). The confidence interval is thus around the quantity *δ*=*D*^∗^−*D*_2_. However, as *D*^∗^ is assumed to have zero variance, *var*(*D*^∗^−*D*_2_)=*var*(*D*_2_); so the width of CI for *δ*=*D*^∗^−*D*_2_ is equivalent to the width of CI for *D*_2_ only.

Thus to estimate *D* in a new study with a confidence interval of half width *w*, we replace ${\sigma _{1}^{2}}$ with 0 in calculation (C), so the number of events required is

(D) $$ e_{2}=\lambda\left(\frac{w}{z_{1-\alpha /2}}\right)^{-2}   $$

Again substituting either *λ*_*s*_ or *λ*_*m*_ we get

(D1) $$ e_{2}=\lambda_{s}\left(\frac{w}{z_{1-\alpha /2}}\right)^{-2}   $$

(D2) $$ e_{2}=\lambda_{m}\left(\frac{w}{z_{1-\alpha /2}}\right)^{-2}   $$

The only limit on *w* when using calculations (D1) and (D2) is that it must be >0.

Note that Eqs. (D) and (B) are equivalent if the power in (B) is 50 % and *α* is two-sided. So, for example, a study designed to estimate *D* with a 95 % confidence interval of half width 0.2 requires the same number of patients as a study designed such that a difference in *D* of *δ*=0.2 from the target value is significant at the (two-sided) 5 % level with 50 % power.

## Results

### Validating the calculations

The calculations were tested for validity using simulation studies. The results of four of these studies (one covering each calculation) are given in the Appendix: simulation studies to test sample size calculations, however further simulations were performed to cover a wide variety of scenarios; these can be found in [23]. We found they all showed the desired power and type I error and this was not affected by random censoring. The calculations using *λ*_*m*_ showed small inaccuracies in power in the simulation studies due to the imperfect nature of estimating *λ* using Eq. (3). These errors in power were of the order of up to 2 % (absolute) when the desired power was 80 %. All the calculations, whether using *λ*_*s*_ or *λ*_*m*_, give the expected power and type I error only if the parameters in the new study are similar to what was expected in the planning stages (either from the previous study or input into the model for *λ*_*m*_). In further simulation studies we found that if the value of *D*, or the censoring proportion, in the new study is larger than it was in the previous study, results in the new study may be less precise than were expected [23]. Equally if the values are smaller in the new study, results may be more precise than planned.

### Implementation of commands in Stata

We have written two commands in Stata to implement the four calculations described here. dsampsi sig calculates the sample sizes required by (B1) and (B2), while dsampsi ci calculates (D1) and (D2). These are available from the author upon request.

### Absolute and relative precision

As *D* increases, or as *cens* increases, the number of events required to retain the same precision increases. This means that if the observed values of these two quantities in the finished study are different to those used in the calculations, the estimate of *D* in the final study will have higher or lower precision than was planned. This inadvertent under- or over-powering is a potential problem in any sample size calculation for survival outcomes, including randomised clinical trials: any divergence from the expected censoring rate or hazard ratio for trial treatment would mean that the original sample size was either too large or too small; however, post-hoc calculations of power are not routinely performed. As the sample sizes output by our calculations are often high, the consequences of this under- or over-powering may be serious for prospectively planned prognostic studies: either many more patients are recruited than were really required, or a study does not meet its aims despite a large sample size.

In an effort to mitigate this problem, we present a pragmatic method to try and minimise the risk of serious under- and over-powering when using (B2) and (D2). Essentially *δ* or *w* are specified as a proportion of *D* rather than as an absolute value, formalising the idea that if *D* turns out to be higher than expected, researchers may be happy with a lower absolute precision than initially proposed.

If we denote by *p* the proportion of the target *D* that we will accept as our *δ* or *w*, then calculations (B2) and (D2) become

(4) $$ e=\lambda_{m}\left(\frac{pD}{zz}\right)^{-2}   $$

(5) $$ e=\lambda_{m}\left(\frac{pD}{z_{1-\alpha /2}}\right)^{-2},   $$

where *D* is the best estimate available.

It is clear that for calculations (4) and (5), as *p* increases the number of events required decreases. Also, it is important to observe that as *D* increases, the number of events required decreases, which means we now have the reverse problem to previously: we *lose* precision if the value of *D* is lower than expected. A straightforward solution is to combine the two approaches in a ‘composite’ sample size; specify both an absolute and a relative precision for *δ* or *w*. For example, for a significance-based calculation we may be happy with precision of either *δ*=0.15 or *p*=10 *%* of *D*, whichever requires the smaller sample size at each value of *D*. We illustrate this strategy further below with real data examples.

### Examples using parameters from a published paper

To illustrate the calculations we recommend as well as our composite sample size proposal, we use as a basis for our examples a paper published in 2008 which compared three existing staging systems for advanced liver cancer [28]. In this study the CLIP prognostic model was found to be most recommended, with *D*=1.01. The standard error of *D* was given as 0.09, and the models were assessed on a dataset of 538 patients with 502 events (7 % censoring).

#### Calculations (B1) and (D1)

Let us first assume that we wish to validate the CLIP model on new data. Our objective is to have assurance of a certain level of performance (discrimination) of the CLIP model, as measured by *D*. Calculations (B1) and (D1) require *λ* to be estimated from the previous dataset; from the reported results in the paper, $\lambda _{s}\,=\,e_{1}{\sigma _{1}^{2}}\,=\,502*0.09^{2}\,=\,4.1$. Note that here we assume that the case mix of the validation study is identical to the development study; if this is not the case then the interpretation of the value of *D* (or, indeed, any other model performance measure) at external validation is more complex [29, 30].

**Fig. 1:**
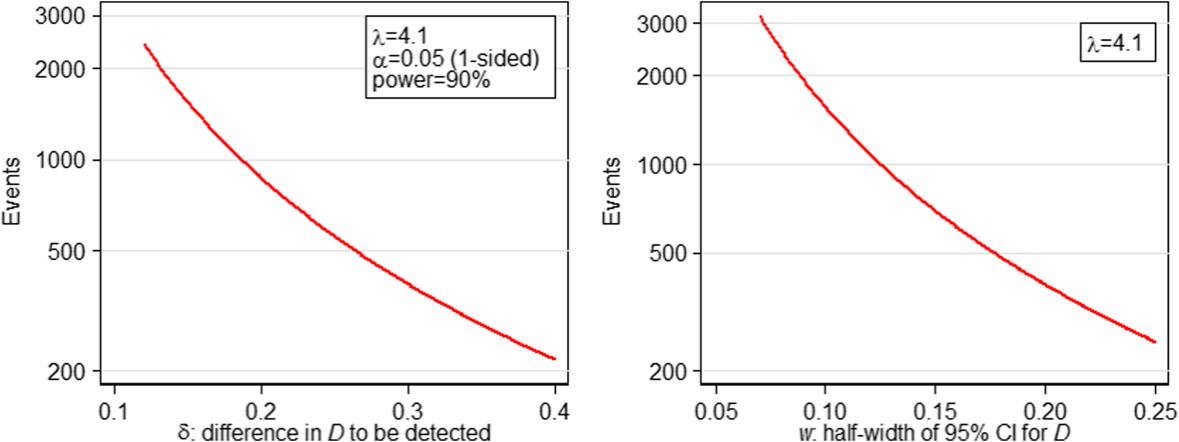
Variation in events required by (left) calculation (B1) vs *δ*; (right) calculation (D1) vs *w*

If we require a significance based non-inferiority study with one-sided *α*=0.05, 90 % power and non-inferiority margin *δ*=0.25, 558 events are required according to calculation (B1). If it is expected that the same censoring proportion will hold in the new study, 601 patients should be recruited. For a study with two-sided *α*=0.05 (all other parameters held equal) 684 events are required. Figure 1 shows how the number of events required by calculation (B1) changes with *δ*, for a one-sided test.

If instead of a significance based calculation we wish to specify the CI for *D* in the new study, then we use (D1). In order that our estimate of *D* in the new study has a 95 % confidence interval with half-width 0.2, we require 391 events. Figure 1 shows the effect of *w* on the sample size calculation (D1).

#### Calculations (B2) and (D2)

Let us now assume that we wish to add a new prognostic factor to the CLIP model which we believe will improve its prognostic ability. As we have no previous study using the proposed model to estimate *λ*_*s*_ from, we can use either (B2) or (D2). These calculations do not require a previous study, just a target value of *D* and the censoring proportion in the dataset to estimate *λ*_*m*_.

To determine a target value of *D*, we note that the paper in question reported *D*=1.01 for the CLIP model, which is equivalent to ${R^{2}_{D}}=19~\%$ [19]. If we believe that the new factor will increase the proportion of variation explained by the model by 10 % (absolute) to ${R^{2}_{D}}=29~\%$, our target value of *D* should be *D*=1.3. If we expect the censoring proportion to be 10 %, slightly higher than the CLIP paper, then using (3) we estimate *λ*_*m*_=4.62.

**Fig. 2:**
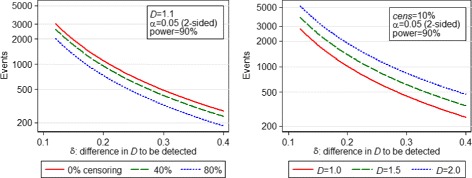
Variation in events required by calculation (B2) vs *δ*, for different values of *cens* (left) and *D* (right)

Under calculation (B2), 633 events are required for a non-inferiority study with one-sided *α*=0.05, 90 % power and non-inferiority margin *δ*=0.25. For the equivalent superiority study with two-sided *α*=0.05, 777 events are required. Figure 2 shows the variation in number of events required by calculation (B2) for a two-sided test vs the *δ* desired, for different values of *D* and *cens*. Note when looking at these graphs that although increasing *cens* decreases the number of events required, the total number of patients required increases.

**Fig. 3:**
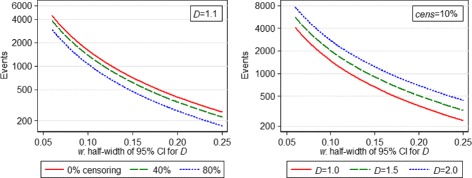
Variation in events required by calculation (D2) vs *w*, for different values of *cens* (left) and *D* (right)

If a CI-based calculation is desired, in order that our estimate of *D* in the new study has a 95 % confidence interval of half-width 0.2, using (D2) we require 444 events (using *λ*_*m*_=4.62). Figure 3 shows how required study size changes with *D*, censoring proportion and *w* according to calculation (D2).

#### Calculating a range of sample sizes

As already mentioned, by using *λ*_*m*_ we can calculate a range of sample sizes by inputting different likely values of *D* and censoring proportion into Eq. (3). We briefly illustrate this using (D2).

We saw above that if we expect *D*=1.3 and 10 % censoring in the new study, to obtain a 95 % CI for *D* with half-width 0.2 we require 444 events (494 patients). If we believe *D* could be as low as 1.1 or as high as 1.5, then inputting these values gives us *λ*_*m*_=4.08 and *λ*=5.24 respectively, which results in sample sizes of 392 and 504 events (436 and 560 patients). If we think the censoring proportion might be as high as 30 % in the new study, then this results in a *λ*_*m*_=4.25 and a sample size of 408 events if *D*=1.3: with 30 % censoring this means 583 patients are required. If *D*=1.5 and *cens*=30 *%* then *λ*_*m*_=4.79 and 461 events are required; 659 patients.

Performing a range of calculations like this may help during study planning, and in assessing whether a retrospective study might be large enough; and may be especially useful when the value of *D* and/or the censoring proportion in the study is uncertain.

#### Combining absolute and relative precision

As mentioned above, a pragmatic method for controlling power when the observed value of *D* is not very certain is to define the desired precision with both absolute and relative limits. We illustrate this idea again using the CLIP paper example.

We return to the scenario outlined above to illustrate calculation (B2), but the same ideas hold for (D2). We once again require a study with two-sided *α*=0.05 and 90 % power. Let us suppose that we are happy with precision of *δ*_*abs*_=0.25 or *p*=20 *%* of *D*, whichever requires the smaller sample size at each value of *D*. The maximum sample size can be calculated by using calculation (B2): making *δ* the absolute value desired (0.25 in this example), and inputting *D*=*δ*_*abs*_/*p* in the equation for *λ*_*m*_ (*D*=0.25/0.2=1.25 in this example). Here this gives *λ*_*m*_=4.47 and a required sample size of 753.

**Fig. 4:**
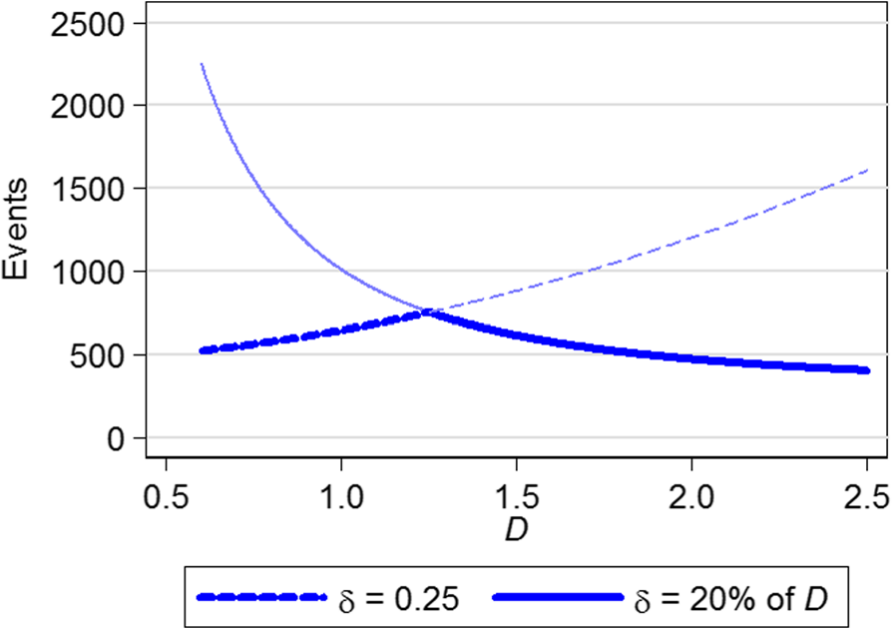
Events required by composite calculation (4) vs *D*, for absolute and relative values of *δ*

Figure 4 shows the sample size curves for the absolute and relative precisions, and the resulting profile for the smallest sample size is shown as a thick line, with a peak at *D*=1.25 where the number of events is 753. With a study of 753 events, if the value of *D* in the new study turns out to be *δ*/*p*=1.25, then the study will have the correct precision. As can be seen in Fig. 4, if the value of *D* is either higher or lower than 1.25 then slightly more patients will have been recruited than strictly required, so the study will have slightly higher than anticipated precision. The precision that will actually be observed with a different value of *D* can be calculated by rearranging (B2) and substituting the new value of *D*. In this example, before the study we would anticipate that if *D*=2, the smallest *δ* detectable with 90 % power and two-sided *α*=5 *%* would be 0.4 (20 % of *D*=2), however with 753 events it is actually 0.32.

### *D* in practice

In order to use calculations (B2) and (D2), researchers must have in mind a target value of *D* so that they can calculate *λ*_*m*_. Although *D* is becoming more commonly reported in prognostic research, it is not yet available for a wide variety of diseases, so it may be difficult to find a suitable value of *D*. For this reason a literature search was carried out to assess how widely *D* is used and to determine its value in various disease areas. The main aim of the search was to show a method by which researchers might find a suitable value of *D* for use in their own work, but additionally the values found in the search may be used as a reference library by users of calculations (B2) and (D2). We also used some of the values collected to develop an equation to convert Harrell’s *c*-index to *D*. The methods and results of the literature search are described in detail elsewhere [23]; we present the main findings here.

The search was divided into two parts: first a search for all reported values of *D*, second a search for a limited number of values of Harrell’s *c*-index. The former resulted in 108 *D* values reported in 34 separate papers; the latter 331 *c* values from 77 papers. We collated a dataset of models from the searches which had both *D* and *c* values reported, and augmented these with values from models developed on publicly available time-to-event datasets (from books and papers). The 294 paired (*D*, *c*) values showed a strong relationship and we modelled this by simply fitting a fractional polynomial to the data, giving Eq. (6) which could be used by researchers to convert a value of *c* to *D* for use in our calculations.

(6) $$ D=5.50(c-0.5)+10.26(c-0.5)^{3}   $$

**Fig. 5:**
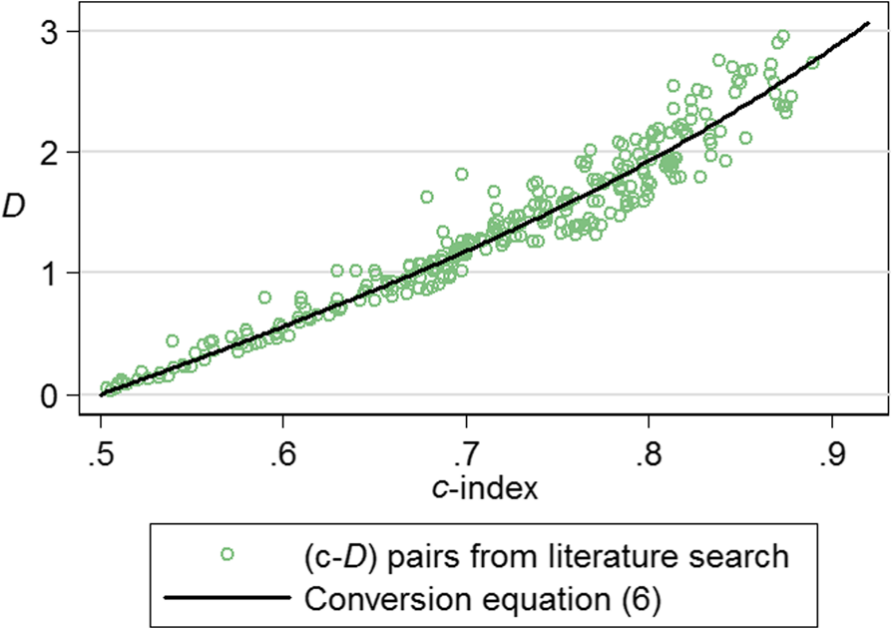
The (*c*,*D*) pairs used to model the relationship between *c* and *D* and the final model Eq. (6)

Figure 5 shows the data used to develop (6) overlaid with the model itself. Table 1 shows various points on the modelled relationship between *D* and *c*.

**Table 1:** The relationship between *c*, *D* and ${R^{2}_{D}}$: selected points

*c*	*D*	${R^{2}_{D}}$		*c*	*D*	${R^{2}_{D}}$
0.50	0.000	0.000		0.72	1.319	0.294
0.52	0.110	0.003		0.74	1.462	0.338
0.54	0.221	0.011		0.76	1.610	0.382
0.56	0.332	0.026		0.78	1.765	0.427
0.58	0.445	0.045		0.80	1.927	0.470
0.60	0.560	0.070		0.82	2.096	0.512
0.62	0.678	0.099		0.84	2.273	0.552
0.64	0.798	0.132		0.86	2.459	0.591
0.66	0.922	0.169		0.88	2.652	0.627
0.68	1.050	0.208		0.90	2.857	0.661
0.70	1.182	0.250		0.92	3.070	0.692

After the obtained *c* values from the literature search were transformed to *D* using (6), the resulting pool of *D* values were explored and grouped by disease area. Ultimately, we obtained 480 values of *D* in total, ranging from 0 to 3.44 and with mean 1.40 (median 1.30). Of these, 296 values were from prognostic models (predicting a disease event in patients who already have the disease of interest) and 184 from risk models (predicting onset of a disease in healthy patients). We found that the mean value of *D* amongst the prognostic models (*D* = 1.30) was slightly lower than for the risk models (*D* = 1.47). A full description of the *D* values collected can be found in [23]. For most diseases only one or two papers were retrieved.

## Discussion

### Recommendations for practice

As argued above, we find that calculations based on (A) and (C) are of limited practical use because of the lower limits on the values of *δ* and *w* that can be detected. Thus the calculations which we find most useful are (B1) and (B2) which are based on significance testing, and (D1) and (D2) which are based on the precision of the estimate of *D* in the new study. It is purely down to the preferences of the researcher as to which type is chosen. Within each type, there are two options depending on whether researchers wish to include information from a previous study in their calculation (B1, D1), or want to (or have no choice but to) choose target values for the parameters (including *D*) instead (B2, D2). The latter option makes it easier to calculate a range of possible sample sizes, as shown in the example above. This may be important if researchers are not very confident about the likely values of *D* that will be seen in the new study, or wish to explore the effects of different censoring proportions. For this reason we would recommend (B2) and (D2) over (B1) and (D1) in most cases. However, if a reliable previous study exists then (B1) and (D1) may be preferred (for example, if researchers are seeking to validate an existing study). If (B1) or (D1) are used, we recommend that a bootstrap estimate of the standard error of *D* is used to calculate *λ*_*s*_ instead of the default estimate provided, as this is likely to underestimate this quantity.

**Fig. 6:**
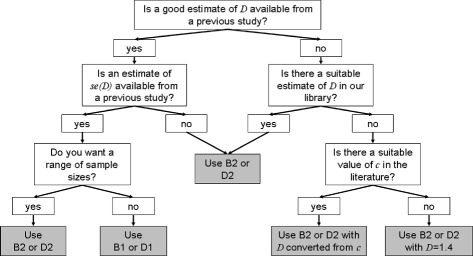
Flowchart to aid decision making

If (B2) or (D2) are chosen, a value of *D* and the censoring proportion for the proposed study must be estimated. Estimating the censoring proportion should be straightforward for researchers but finding a suitable value of *D* may be more problematic. If an appropriate value cannot be found in the library of values presented in [23], we recommend that researchers search literature for a suitable *c*-index value and convert this instead using (6). The question of what is a ‘suitable’ value of either *c* or *D*, in terms of how similar the study population, methods, model and other aspects must be, is difficult to answer and we do not attempt to give a solution here. In the absence of any guidance whatsoever as to a suitable value of *D*, we suggest using a value of *D*=1.4, the mean value of *D* across the large number of prognostic models collated here. We give a decision-making flowchart in Fig. 6 to help potential users of our method determine which calculations can be used in their situation.

Although the sample sizes output by the calculations tend to be large, we have given some suggestions on how study size can be managed, for example by considering precision as a proportion of the measure of interest, rather than (or as well as) a fixed value. We recommend using this method to prevent inadvertent loss of precision due to uncertainty around the estimated value of *D* when using (B2) or (D2). However it is worth noting that this under- or over-powering is a potential problem in any scenario, including randomised clinical trials.

## Conclusions

Prognostic studies using time-to-event data are often performed and appear frequently in medical literature. In general, the aim of such studies is to develop a multivariable model to predict the outcome of interest, and they often use time-to-event data analysed with the Cox proportional hazards model. Many prognostic studies are performed with retrospective data and often without reference to sample size calculations [2], suggesting that obtaining reliable results from such studies may often be a matter of chance.

The main sample size guidance available to and used by researchers developing prognostic survival models is the events per variable (EPV) calculation with a lower limit of 10 EPV usually quoted; however, this idea is based on just two limited simulation studies. These studies concentrated on the significance of model coefficients, which is of secondary importance in a prognostic model to be used for outcome prediction. In this paper we have presented some sample size calculations based instead on the discrimination ability of a survival model, quantified by Royston and Sauerbrei’s *D* statistic. We have also given some suggestions and methods for improving the practical use of the calculations in research.

Due to the novel nature of the methods presented in this paper, there are limitations to the work described here and further avenues yet to be explored. In particular, we note that the sample size calculations presented here pay no attention to the number of variables to be explored. From previous work we know that the number of candidate variables for a model can have an effect on the estimate of *D* in some situations [23]. If a model is developed using an automatic variable selection method and then validated in the same dataset, then increasing the number of candidate variables increases the optimism present in the estimate of *D*; however, we have not covered this issue here. Additionally, we acknowledge that changes in case mix between datasets can add complexity to defining improvement in the prognostic performance of a model, whether *D* or some other performance measure is used. The methods introduced in [29] may offer a solution to this problem but it is too early to say; in this paper we have made the assumption that the distributions of covariates are comparable between datasets used for model development and validation purposes.

We hope that these calculations, and the guidance provided for their use, will help improve the quality of prognostic research. As well as being used to provide sample sizes for prospective studies in time-to-event data, they can also be used for retrospective research; either to give the required sample size before suitable existing data is sought, or to calculate the likely precision of results where a dataset has already been chosen. At the very least we hope that the existence of these calculations will encourage researchers to consider the issue of sample size as a matter of course when developing or validating prognostic multivariable survival models.

## Appendix: simulation studies to test sample size calculations

The sample size calculations were tested using simulation, to check that they provided the desired power and *α*, or the desired confidence interval width.

We simulated time-to-event data from an exponential distribution, with baseline cumulative hazard function *H*_0_, using the method described by Bender et al. [31]. The survival time for the proportion hazards (PH) model with regression coefficients (log hazard ratios) *β* and covariate vector *X* was simulated using

(7) $$ T_{s}=H_{0}^{-1}[-\log (U)\exp (-\beta^{\prime }X)]  $$

where *U*∼*U*[0,1]. Since simulating a full multivariable vector is complex both computationally and in terms of interpretation, we instead used a surrogate scalar *X*. *X* was simulated as ∼*N*(0,1), and the value of *β* fixed, so that the resulting prognostic index *βX* was also normal. In a dataset simulated this way, *D*=*βκ* [23].

We simulated random non-informative right-censoring using the same method to obtain an exponentially distributed censoring time *T*_*c*_ for each patient; note *T*_*c*_ were not dependent on *X*. Records where *T*_*c*_<*T*_*s*_ were considered censored at time *T*_*c*_. The desired censoring proportion was achieved by changing the baseline hazard.

Throughout our simulations we wished to use datasets with an exact number of events and censoring proportion. To obtain a dataset with exactly *e*_1_ events and exact censoring proportion *cens*, we first generated a dataset with $2(\frac {e_{1}}{1-cens})$ records and approximate censoring proportion *cens*. We then simply randomly selected *e*_1_ records ending in failure, and $\frac {e_{1}}{1-cens}-e_{1}$ censored records, to form the final dataset.

The variance or standard error of *D* was obtained by bootstrap whenever required.

### Calculations (B1) and (B2)

For (B1) the first step of the simulation is to generate a ‘first’ study with *e*_1_/(1−*cens*) records and exactly *e*_1_ events. This dataset is bootstrapped to obtain ${\sigma _{1}^{2}}$, the variance of *D*, and then *λ*_*s*_ is calculated from this quantity and *e*_1_. For (B2) the first step is to calculate *λ*_*m*_ from Eq. (3) with the desired estimates of *D* and cens.

The next steps are common to both (B1) and (B2) once *e*_2_ is calculated. Datasets of the required size are generated separately under the null and alternative hypotheses, and bootstrapped to obtain *se*(*D*). The whole procedure is repeated 2000 times for each combination of parameters varied (*D*, power, *δ* and cens), and test statistics calculated to determine if the number of events *e*_2_ gives the required power and type 1 error. A selection of results is given in Table 2. For (B1), this table shows the results for *e*_1_=750; the simulations were repeated for *e*_1_=1500 and showed very similar results but these are not presented here.

**Table 2:** Results of simulation study to test (B1) and (B2)

Simulation parameters						Observed (B1)				Observed (B2)		
*β*	*D*	power	*δ*	cens		*e*_2_	% type 1 (se)	% power (se)		*e*_2_	% type 1 (se)	% power (se)
1.0	1.6	80 %	0.4	0		222	5.5 (0.51)	81.7 (0.86)		222	5.0 (0.49)	81.5 (0.87)
				80		141	5.6 (0.51)	80.8 (0.88)		133	5.1 (0.49)	79.5 (0.90)
2.0	3.2	90 %	0.5	0		495	4.0 (0.44)	89.6 (0.68)		483	4.0 (0.44)	88.1 (0.73)
				80		286	4.8 (0.48)	92.1 (0.60)		291	4.4 (0.46)	92.2 (0.60)

### Calculations (D1) and (D2)

As for (B1), the first step of the simulation study for (D1) is to generate a ‘first study’ to provide values of *e*_1_ and ${\sigma _{1}^{2}}$ for the calculation of *λ*_*s*_.

For (D2) *λ*_*m*_ is calculated using Eq. (3). For both (D1) and (D2), once *e*_2_ has been calculated, a dataset with the required number of events and censoring proportion is simulated and *D* calculated. This was repeated 2000 times for each combination of parameters. The proportion of repetitions for which the estimate of *D* is within *w* of the input *D*=*βκ* gives the % CI which has width ±*w*. This should approximate 1−*α*, if the sample size calculation and estimation of *λ* are correct. A selection of results is given in Table 3. For (D1), this table shows the results for *e*_1_=750; the simulations were repeated for *e*_1_=1500 and showed very similar results but these are not presented here.

**Table 3:** Results of simulation study to test (D1) and (D2)

Simulation parameters					Observed (D1) (95 % CI)			Observed (D2) (95 % CI)	
*β*	*D*	*w*	cens		*e*_2_	% of *D* (se) within *β* *κ*±*w*		*e*_2_	% of *D* (se) within *β* *κ*±*w*
1.0	1.6	0.2	0		553	94.7 (0.50)		550	94.8 (0.50)
			80		348	94.6 (0.51)		331	94.4 (0.51)
2.0	3.2	0.3	0		616	94.6 (0.51)		602	94.4 (0.51)
			80		356	94.7 (0.50)		363	94.4 (0.52)
